# Should Trust Be Stressed? General Trust and Proactive Coping as Buffers to Perceived Stress

**DOI:** 10.3389/fpsyg.2020.554962

**Published:** 2020-11-13

**Authors:** Anders Carlander, Lars-Olof Johansson

**Affiliations:** ^1^SOM Institute, University of Gothenburg, Gothenburg, Sweden; ^2^Wallenberg Centre for Molecular and Translational Medicine, University of Gothenburg, Gothenburg, Sweden; ^3^Department of Psychology, University of Gothenburg, Gothenburg, Sweden

**Keywords:** stress, personality, intolerance of uncertainty, coping, trust

## Abstract

Stress is becoming an increasingly important public health concern. Assuming that individual levels of trust and coping can buffer psychological stress, we explore validated measures of general trust [General Trust Scale (GTS)], proactive coping [Proactive Coping Inventory (PCI)], jointly with personality [Honesty-Humility, Emotionality, Extraversion, Agreeableness, Conscientiousness, and Openness to experience (HEXACO)], and intolerance of uncertainty (IUS), as predictors of perceived stress [Perceived Stress Scale (PSS)]. Data were collected from Qualtrics research panels using quota sampling to obtain two representative American community samples. The assumed alleviating effects of GTS and PCI on PSS remained but were attenuated when modeled jointly with HEXACO, IUS, and socio-economic background variables [socioeconomic status (SES)] in hierarchical regressions. In Study 1 (*N* = 1,213), SES explained 19% and HEXACO explained 29% of the variance in PSS. Introducing IUS and GTS added significant but small portions of explained variance. In Study 2 (*N* = 1,090), after controlling for SES which explained 18% of the variance, IUS explained an additional 18% of the variance in PSS. Adding GTS to the model showed modest contributions whereas PCI added 9% of explained variance in the final hierarchical step. The findings highlight that GTS and PCI remain important variables even after controlling well-known factors such as personality and ability to tolerate uncertainty. However, given the weak effects of GTS, to consider trust as a remedy for stress may be of limited use in clinical practice since it could potentially be explained largely as a proxy for a beneficial combination of personality, coping, and socioeconomic background.

## Introduction

Psychological stress is an increasingly important issue (APA, 2020) with potentially important implications for people’s health (Schneiderman et al., 2005; Folkman and Nathan, 2011), and cognitive function (Lupien et al., 2009; Pechtel and Pizzagalli, 2011) and may be seen as a risk factor for psychopathology (Charles et al., 2013; Salim, 2014). In understanding what may drive or alleviate psychological stress, individual factors such as personality and coping strategies are important (Carver and Connor-Smith, 2010). Recently, the health benefits of trust have attracted attention not only in psychology but also in the fields of medicine and public health (Kawachi et al., 2008; Giordano and Lindström, 2016; Giordano et al., 2019). As health-promoting factors, the efficacy and relevance of related contextual effects and similar constructs to general trust also need to be evaluated (Shiell et al., 2020).

Consistent with the stress-buffering hypothesis of social support (Cohen and Wills, 1985), we posit that trust may function as a coping mechanism when people face stressors. Measures of trust and coping have however rarely been modeled together with more dispositional factors such as personality and intolerance for uncertainty. In line with Shiell et al. (2020), we recognize a need to investigate and disentangle the alleged stress-buffering effects of trust. We pursue this by testing how measures of general trust and proactive coping relate to perceived stress over and above what measures of personality and intolerance of uncertainty (IU) explain.

Psychological stress, commonly thought of as an interplay between cognitive and biological processes (Schneiderman et al., 2005), may be seen as an outcome of appraising threatening events that can result in stress reactions if and when coping resources prove to be inadequate (Lazarus and Folkman, 1984). Stress responses tend to vary with personality traits (Penley and Tomaka, 2002), often in conjunction with self-efficacy (Ebstrup et al., 2011). Psychological stress can be assessed using the Perceived Stress Scale (PSS), which measures the respondent’s subjective stress level within a certain timeframe (Cohen et al., 1983).

Stress and negative emotions are furthermore shown to be positively associated with the Neuroticism trait and negatively related to the Extraversion, Agreeableness, Conscientiousness, and Openness traits from the Five Factor Model (FFM) of personality (Costa and McCrae, 1992). Moreover, previous research shows that different psychopathological conditions, including depression, anxiety disorders, phobias, and substance, use relate to higher Neuroticism and lower Extraversion and Conscientiousness (Kotov et al., 2010). It is generally believed that especially the Neuroticism trait will influence how individuals appraise threats and stressors, something which in turn may increase the risk of detrimental responses (Carver and Connor-Smith, 2010; Ebstrup et al., 2011). In addition, while Extraversion is related to more engaged coping strategies such as problem-solving and use of social support, Neuroticism is associated with disengaged coping strategies such as denial and substance use (Carver and Connor-Smith, 2010; Coulston et al., 2013). Given this, we believe that certain combinations of personality traits may influence and explain both how people appraise stressful events and how they engage their available coping resources.

Similar to the more common FFM (Costa and McCrae, 1992), the HEXACO personality inventory (which is used in the present research) is a trait model consisting of the six personality factors, Honesty-Humility (H), Emotionality (E), Extraversion (X), Agreeableness (A), Conscientiousness (C), and Openness (O) to experience (Lee and Ashton, 2004). A benefit of HEXACO is that the Honesty-Humility factor can be seen as the inverse to the shared variance of the dark triad of psychopathy, Machiavellianism, and narcissism (Lee and Ashton, 2005, 2014), which means that it functions well as a theoretically coherent and empirically independent complement to the more common FFM model. The measures of Honesty-Humility and Agreeableness are sometimes referred to as prosocial traits that may be positively linked to generosity and reciprocity (Zhao et al., 2016). Compared with the FFM model the additional Honesty-Humility factor offers some insights, particularly when studying socially relevant behaviors and attitudes.

Due to the necessity of anticipating future events, uncertainty requires an increased cognitive load. Previous research identifies individual differences in how well people tolerate uncertainty and ambiguity (Birrell et al., 2011). Originally developed by Freeston et al. (1994), IU was proposed as an important mechanism and antecedent to worry, seen as a trans-diagnostic construct, distinct from personality (McEvoy and Mahoney, 2012), and believed to be linked to several common psychopathologies including anxiety and mood disorders (Gentes and Ruscio, 2011; Carleton, 2016). Generally, IU deals with subjective notions of the unpredictability of negative events that can incur cognitive (aversion), emotional (anger, discomfort), and behavioral (avoidance, paralysis) responses, linked to both internalizing and externalizing psychopathology. IU can be assessed by the Intolerance of Uncertainty Scale (IUS, Freeston et al., 1994), which we use as a predictor in the present research. Higher scores on IUS is in general related to mood and anxiety disorders (Carleton et al., 2012), and the underlying factors of IU have been suggested to be linked to different behavioral outcomes in the face of uncertainty (see Birrell et al., 2011 for a review). The IUS and PSS measures have been reported to be positively related (Crum et al., 2013), something which may be explained by research showing that subjective uncertainty estimates induce subjective and physiological stress responses (De Berker et al., 2016). This implies that a higher dispositional sensitivity to uncertainty may lead to higher stress levels.

In relation to uncertainty, the concept of trust is often defined as a belief or intention to accept vulnerability based on a positive expectation regarding the intentions and behaviors of other people (Van Lange, 2015). Although trust has been researched in many scientific domains, the most common approach is to distinguish between trustworthiness (judgment of someone’s ability, benevolence, integrity, etc.), trust propensity (the dispositional tendency to depend on others), and trust (the intention and behavior of entrusting someone; see Colquitt et al., 2007 for a meta-analysis). Furthermore, from a psychological perspective reasons for trusting include individual differences in disposition and personality, intergroup processes, and cognitive expectations of outcomes (Evans and Krueger, 2009). In other words, intention to trust may depend on a natural inclination to trust if there is a group or social norm encouraging trust and a beneficial outcome associated with trusting someone. The idea that trust can at least partly be seen as a dispositional characteristic (Rotter, 1971) or a personality trait (Costa and McCrae, 1992) is not new. Trust has largely been assumed to be an adaptive result of genes, social upbringing, and successful or aversive experiences (Glanville and Paxton, 2007; Van Lange, 2015). It is therefore not surprising that trust has been reported to correlate positively with Extraversion and Agreeableness traits (Hiraishi et al., 2008). In addition, while Conscientiousness and Openness may be positively related to trustworthiness, Neuroticism has been found to correlate negatively with trust (Evans and Revelle, 2008). Moreover, an inverse relationship between trust and stress is reported in studies of the associated hormones oxytocin (trust) and cortisol (stress, Takahashi et al., 2005; Cardoso et al., 2013; McQuaid et al., 2016), and of how stress-induced participants seek out prosocial interactions including trust (von Dawans et al., 2012; Raposa et al., 2015). Similarly, higher stress may lead to less trusting decision-making (FeldmanHall et al., 2015). In addition, it has been confirmed in both neuroimaging experiments (Yanagisawa et al., 2011) and experiments that when faced with stressors and threats people may upregulate their level of trust (Koranyi and Rothermund, 2012), possibly as a way of coping. Positive relationships have also found between trust and active appraisal and coping strategies (Buchwald, 2003; Wilson and Darke, 2012).

In trust research, another approach is to observe levels of trust in general within and between social groups. Such general trust (sometimes referred to as social capital) is generally considered important for society at large. It has been reported to be associated with general well-being (Carl and Billari, 2014), well-functioning democracies (Uslaner, 2002), economic growth (Guiso et al., 2004), cooperation (King-Casas et al., 2005), intelligence (Hooghe et al., 2012), and health (Kawachi et al., 2008; Giordano and Lindström, 2016; Giordano et al., 2019). Trust is thus associated with several beneficial outcomes that may function as buffers to adverse stressors. Resilience to stress has been described by Folkman and Lazarus (1985) as a dynamic coping process that involves efforts to face external threats and demands. Research on coping has traditionally focused on reactive strategies, e.g., “problem-focused coping” and “emotion-focused coping” (Lazarus and Folkman, 1984). Other approaches such as proactive and preventive coping (e.g., Schwarzer and Taubert, 2002) are more goal-oriented in creating a mental preparedness for potentially stressful events. Proactive coping is assumed to promote a positive mood and an enhanced focus on future events that may alleviate psychopathological outcomes (Greenglass and Fiksenbaum, 2009). While most measurement instruments regard coping as a reactive process (e.g., Kato, 2013), the Proactive Coping Inventory (PCI), which is used in the present research (Greenglass and Fiksenbaum, 2009), represents a more forward-oriented outlook, measuring characteristics such as self-regulation and goal attainment. The PCI measure has been found to also correlate with the personality traits measured in the FFM of personality (Costa and McCrae, 1992); in particular, Extraversion and Conscientiousness appear to be positively related to PCI, whereas Neuroticism shows a negative relationship (Straud et al., 2015).

As reviewed above, while there is some evidence that general trust is beneficial for health (Kawachi et al., 2008; Giordano and Lindström, 2016; Giordano et al., 2019), there is a need to also investigate contextual effects and similar constructs of trust in order to explain and evaluate the efficacy and relevance of trust as a health-promoting factor (Shiell et al., 2020). In line with the stress-buffering hypothesis of general trust (Cohen and Wills, 1985; McQuaid et al., 2016), we apply an eclectic approach, in order to deepen our understanding of the health-promoting effects of general trust (Shiell et al., 2020). We do this by examining how individual differences in general trust, proactive coping, personality, and IU jointly relate to perceived stress. In two representative American community samples, we test the statistical relations between the measurements of these constructs, while controlling for background variables (age, gender, education, and income). To our knowledge, few studies have investigated how such constructs jointly relate to, drive and possibly alleviate perceived stress in general population samples. We question whether a partly dispositional measure of general trust (GTS) and a more context sensitive measure of proactive coping (PCI) can explain variance in perceived stress (PSS) over and above what background variables [socioeconomic status (SES)], personality (HEXACO), and intolerance of uncertainty (IUS) can explain.

## Study 1

### Participants and Procedure

Participants partaking in Study 1 (*N* = 1,213) were recruited from Qualtrics Research Panels in June 2018. The design of the study was a cross-sectional survey programmed in the online survey platform Qualtrics XM. Qualtrics provides access to a pool of online panels that can be recruited to partake in research studies. This type of data collection method is becoming increasingly popular and the data quality from the Qualtrics panel seems to be on par with data from conventional data collection methods (e.g., Walter et al., 2019). A Qualtrics project manager invited panel participants as needed using quota sampling in order to achieve a sample of participants residing in the United States where the sample had the same proportion as the American population based on gender (51.2% female), age (18–24 = 12.7%, 25–34 = 17.8%, 35–44 = 16.9%, 45–54 = 18.1%, 55–64 = 16.2%, 65+ = 18.2%), education (bachelor’s degree or higher = 33.5%), and income (US median income or less = 44.1%). This sampling procedure entails a non-probabilistic stratification, in that participants are continually invited until the pre-defined quota, for example between males and females, is met. There were no missing data in the dataset since we used firstly response requests and secondly forced responses for each question. The identity of the participants is not known to us but technically the identities of the participants may be seen as a pseudonymized form of de-identification since Qualtrics has a theoretical possibility to link a response ID with a living person.

### Measures

The PSS scale has been validated in a number of languages and cultures (Lee, 2012) and is shown to be related to biological stress markers (Epel et al., 2004). The PSS originally consisted of 14 items (PSS-14) measuring perceived stress but was later reduced to 10 items (PSS-10) due to improved psychometric properties (Cohen and Williamson, 1988) and is therefore recommended for both practice and research (Lee, 2012). The PSS-14 (Cohen et al., 1983) contains 14 items that ask respondents about their feelings and thoughts during the last month. Each statement is phrased using *how often* and is rated on a five-point scale ranging from 0 (“never”) to 4 (“very often,” e.g., “In the last month, how often have you felt nervous and ‘stressed’?”). The PSS-14 score is commonly obtained by summing all items to a total perceived stress score. We collected data for the 14 items but we used the 10-item version (PSS-10; Cohen and Williamson, 1988) in the analyses.

Intolerance of Uncertainty Scale was developed as an instrument comprising 27 items measuring dispositional sensitivity for uncertain events (Freeston et al., 1994), and was later translated (Buhr and Dugas, 2002) and shortened to a 12-item version (Carleton et al., 2007) with reliable psychometric properties (Hale et al., 2016). The IUS-12 (Carleton et al., 2007) consists of 12 statements about subjective responses to uncertain events. The IUS-12 comprises two factors: prospective anxiety (e.g., “I always want to know what the future has in store for me”) and inhibitory anxiety (e.g., “When it’s time to act, uncertainty paralyzes me”). Each statement is assessed using a five-point rating scale, ranging from 1 (“Not at all characteristic of me”) to 5 (“Entirely characteristic of me”). The scoring of IUS-12 is usually assessed using the total or the sum of the statements pertaining to each of the two factors.

The HEXACO-60 short personality inventory (Ashton and Lee, 2009) assesses the six dimensions of the HEXACO model of personality: Honesty-Humility (H), Emotionality (E), Extraversion (X), Agreeableness (A), Conscientiousness (C), and Openness to experience (O). Each dimension contains 10 statements taken from the original HEXACO-PI-R (e.g., “If I want something from someone, I will laugh at that person’s worst jokes”). Each statement is rated on a five-point scale ranging from 1 (“strongly disagree”) to 5 (“strongly agree”).

General trust is often measured in surveys such as the American General Social Survey (GSS) using a single-item question. Yamagishi and Yamagishi (1994) proposed a six-item scale, the GTS, based on several other scales. GTS has been validated across domains and in different languages, and the most recent version is a revised and shortened five-item scale, which is used in the present study (Yamagishi et al., 2015). Each statement of the GTS scale (e.g., “Generally, I trust others”) is rated on a five-point scale ranging from 1 (“completely disagree”) to 5 (“completely agree”).

### Assumptions and Data Analytic Plan

All included constructs are computed as aggregate variables according to each instrument’s scoring. We provide descriptive statistics for each variable including Mean, Standard Deviation, Skewness, Kurtosis, Cronbach’s *α*, and a Pearson correlation table, showing how each variable is related in terms of bi-variate linear correlation.

Broadly, we assume that the instruments used as predictors are confounded and thus may overlap statistically in explaining psychological stress (PSS). A set of more specific assumptions will guide how we approach the data in the following steps. Trait personality theory consists of broader and narrower constructs often hierarchically ordered (e.g., Soto and John, 2017). It is widely accepted that higher-order constructs and processes develop or manifest into lower-order traits followed by even lower-order specific actions and strategies. Hierarchically higher-order traits are less context-dependent and more related to neurobiological underpinnings, whereas lower-order facets are more context-dependent in relation to specific situations and behaviors (DeYoung, 2010, 2013). We will apply a hierarchical approach *between*, as opposed to *within*, different constructs when modeling the data.

While Sexton et al. (2003) have modeled personality traits as a higher-order construct compared to IUS and PSS, Hiraishi et al. (2008) specify general trust as a lower-order construct compared to personality. We, therefore, assume that HEXACO is a higher-order construct compared to IUS, GTS, and PSS. We accordingly model them from higher to lower order in a hierarchical multiple regression analysis (e.g., Tabachnick and Fidell, 2013), in that a higher-order construct will precede a lower-order construct in the equation. The control variables of SES are however entered first.

We expect IUS and Emotionality (E) to be positively related to PSS, whereas the other personality traits likely are negatively related to PSS (e.g., Carver and Connor-Smith, 2010). Further, we expect that the bi-variate relationship between GTS and PSS will be weaker when modeled hierarchically together with the higher-order constructs of HEXACO and IUS. We furthermore expect that controlling for SES (Age, Gender, Education, and Income) will influence the effects of IUS, HEXACO, and GTS on PSS based on the assumption that education and income also have the potential to alleviate perceived stress (Redmond et al., 2013; Algren et al., 2018).

### Results

As may be seen in Table 1, PSS shows, as expected, positive significant correlations with IUS and Emotionality (E). In addition, Extraversion (X) shows a stronger negative correlation to PSS compared to Honesty-Humility (H), Agreeableness (A), and Conscientiousness (C). Furthermore, Openness (O) shows a weak but significant correlation with PSS, while the other pro-social personality traits (H, A, C) and GTS show negative correlations.

**Table 1:** Means (*M*), standard deviation (SD), skewness (Sk), kurtosis (Ku) and Pearson correlations of study variables.

Variables	*M*	*SD*	Sk	Ku	1	2	3	4	5	6	7	8	9
1. PSS-10	1.72	0.74	0.11	3.07	**0.89**								
2. IUS-12	2.67	0.74	0.40	2.87	0.47^***^	**0.87**							
3. H	3.55	0.62	−0.09	2.76	−0.25^***^	−0.22^***^	**0.73**						
4. E	3.18	0.61	−0.11	3.41	0.48^***^	0.43^***^	−0.01	**0.75**					
5. X	3.18	0.67	−0.40	3.38	−0.53^***^	−0.33^***^	0.10^***^	−0.35^***^	**0.81**				
6. A	3.22	0.59	−0.22	3.36	−0.36^***^	−0.23^***^	0.33^***^	−0.16^***^	0.36^***^	**0.75**			
7. C	3.68	0.59	−0.21	2.61	−0.34^***^	−0.15^***^	0.40^***^	−0.12^***^	0.33^***^	0.26^***^	**0.78**		
8. O	3.33	0.66	−0.12	2.96	−0.08^**^	−0.11^***^	0.06^*^	−0.10^**^	0.31^***^	0.19^***^	0.27^***^	**0.78**	
9. GTS	3.27	0.82	−0.43	2.89	−0.36^***^	−0.11	0.08^**^	−0.14^***^	0.38^***^	0.29^***^	0.13^***^	0.13^***^	**0.90**

Internal consistency reliabilities (Cronbach’s *α*) in bold in the main diagonal. An extended version of the correlations including subscales of IUS-12 as well as factors and facets of HEXACO is available in the Supplementary Material. PSS-10, Perceived Stress Scale (short 10-tem version); IUS-12, Intolerance of Uncertainty Scale (short 12-item version); H, Honesty Humility (60-item HEXACO-PI-R (HEXACO-60); E, Emotionality (60-item HEXACO-PI-R (HEXACO-60); X, Extraversion (60-item HEXACO-PI-R (HEXACO-60); A, Agreeableness (60-item HEXACO-PI-R (HEXACO-60); C, Conscientiousness (60-item HEXACO-PI-R (HEXACO-60); O, Openness (60-item HEXACO-PI-R (HEXACO-60); GTS, General Trust Scale (revised 5-item version).

* *p* < 0.05;

** *p* < 0.01;

*** *p* < 0.001.

In order to examine the expectations described above, we conducted a four-step hierarchical regression analysis. As may be seen in Table 2, in the first step, four SES-variables (Age, Gender, Education, and Income) are entered together accounting for 19% of the variance in PSS. All SES-variables show unique and significant associations in that higher age, higher income, and higher education as well as male gender are all associated with lower PSS. The second step reveals that HEXACO adds 29% of the explained variance in PSS. Jointly with SES, nearly half of the variance in PSS is now explained. Each factor in the HEXACO model is a significant predictor of PSS, where E and O indicate positive relationships and the other factors indicate negative relationships to PSS. As expected, Emotionality and Extraversion show the strongest associations. In the third step, the inclusion of IUS shows a significant and positive relationship with PSS and adds 3% of the uniquely explained variance with slightly attenuated coefficients of Emotionality and Extraversion. When GTS is included in the fourth and final step, the explained variance is only marginally improved. Still, GTS shows a unique and significant negative relation to PSS when all other variables are controlled for.

**Table 2:** Hierarchical multiple regression model testing effects on the Perceived Stress Scale (PSS-10).

Variables	Model^a^							
	1		2		3		4	
	*β*	*SE*	*β*	*SE*	*β*	*SE*	*β*	*SE*
H			−0.09^***^	0.03	−0.06^*^	0.03	−0.06^**^	0.03
E			0.31^***^	0.03	0.23^***^	0.03	0.24^***^	0.03
X			−0.30^***^	0.03	−0.26^***^	0.03	−0.23^***^	0.03
A			−0.13^***^	0.03	−0.12^***^	0.03	−0.10^***^	0.03
C			−0.12^***^	0.03	−0.13^***^	0.03	−0.14^***^	0.03
O			0.11^***^	0.03	0.11^***^	0.02	0.11^***^	0.02
IUS-12					0.19^***^	0.02	0.20^***^	0.02
GTS							−0.13^***^	0.02
Age	−0.28^***^	0.01	−0.15^***^	0.01	−0.12^***^	0.01	−0.11^***^	0.01
Gender^b^	0.12^***^	0.04	−0.01	0.04	0.01	0.04	0.01	0.03
Education	−0.09^**^	0.01	−0.02	0.01	−0.03	0.01	−0.01	0.01
Income	−0.15^***^	0.01	−0.08^**^	0.01	−0.08^**^	0.01	−0.08^**^	0.01
*_Adj_R*^2^	0.19^***^		0.48^***^		0.51^***^		0.52^***^	
*Δ_Adj_R*^2^			0.29^***^		0.03^***^		0.01^***^	
*F*	71.52^***^		114.78^***^		116.08^***^		112.42^***^	

PSS-10, Perceived Stress Scale (short 10-tem version); IUS-12, Intolerance of Uncertainty Scale (short 12-item version); H, Honesty Humility (60-item HEXACO-PI-R (HEXACO-60); E, Emotionality (60-item HEXACO-PI-R (HEXACO-60); X, Extraversion (60-item HEXACO-PI-R (HEXACO-60); A, Agreeableness (60-item HEXACO-PI-R (HEXACO-60); C, Conscientiousness (60-item HEXACO-PI-R (HEXACO-60); O, Openness (60-item HEXACO-PI-R (HEXACO-60); GTS, General Trust Scale (revised 5-item version).

a Perceived Stress Scale (PSS-10) modeled as dependent variable.

b Female = 1.

* *p* < 0.05;

** *p* < 0.01;

*** *p* < 0.001.

### Discussion

These results indicate firstly, that the bi-variate relationships displayed in Table 1 can be disentangled and to some degree explained using a hierarchical regression model. Secondly, the assumed higher-order HEXACO construct (Sexton et al., 2003) contributes the most toward explaining PSS, especially the traits of Emotionality (cf. Neuroticism) and Extraversion, which is in line with previous research (Carver and Connor-Smith, 2010; Kotov et al., 2010). Furthermore, although IUS is commonly related to worry and anxiety (Carleton, 2016), the results show that IUS and PSS are associated, as have been reported in other studies as well (Crum et al., 2013). According to McEvoy and Mahoney (2012), the construct of IU should theoretically be seen as distinct from personality and more specifically the trait of Neuroticism. However, the results above indicate that the relationship between IUS and PSS is attenuated when modeled together with HEXACO. Moreover, IUS remains a stronger predictor of PSS compared to GTS, which may support the notion that IU is a higher ordered construct compared to trust (Hiraishi et al., 2008). Although the coefficients are relatively small in the regression compared to the bi-variate correlations, IUS and GTS add significantly to the explained variance of PSS, even after controlling for strong predictors such as personality and socioeconomic background. We, therefore, see the observed effects of IU and general trust as important predictors of psychological stress.

Since we assumed the tested constructs to be inter-correlated it should be noted that each incremental step in the hierarchical regression was expected to increase explained variance. It should also be acknowledged that predictors entered first in a multiple linear regression which generally tend to account for the largest share of variance. It is, therefore important to consider the possible hierarchical order between the constructs and we have provided a rationale for why higher-order constructs such as personality to some extent could account for the variance in lower-order constructs like IU or trust in predicting perceived stress.

## Study 2

In Study 2, we move forward by once again testing the potentially alleviating effects of trust, this time however without HEXACO in the model. Instead, we decided to include a multi-dimensional but lower-order measure of proactive coping, PCI (see Section Measures and Assumptions and Data Analytic Plan for details).

### Participants and Procedure

Participants partaking in Study 2 (*N* = 1,090) were recruited from Qualtrics Research Panels in November 2018. The procedure for Study 2 was practically identical as in Study 1. The study was a cross-sectional design using quota sampling in order to achieve a sample of participants residing in the United States that had the same proportions as the American population based on gender (53.9% female), age (18–24 = 12.3%, 25–34 = 18.7%, 35–44 = 17.7%, 45–54 = 18.3%, 55–64 = 15.9%, 65+ = 17.1%), education (bachelor’s degree or higher = 33.3%), and income (US median income or less = 45.9%).

### Measures

Similar to Study 1, the participants responded to the PSS-14 (Cohen et al., 1983), the IUS-12 (Carleton et al., 2007), and the GTS (Yamagishi et al., 2015; see Section Measures in Study 1). We also included the PCI measure (Greenglass et al., 1999) consisting of 55 items using a four-point rating scale ranging from 1 (“Not true at all”) to 4 (“completely true”). Greenglass et al. (1999) developed the multidimensional PCI consisting of the seven subscales: Proactive Coping Scale (PCS), Reflective Coping Scale (RCS), Strategic Planning Scale (SPS), Preventive Coping Scale (PrCS), Instrumental Support Seeking Scale (ISSS), Emotional Support Seeking Scale (EMSS), and Avoidance Coping Scale (ACS). These subscales partly overlap and their precise factor structures have been questioned (Roesch et al., 2009), but the two fundamental factors of proactive (PCS) and preventive coping (PrCS) seem to represent distinct and unidimensional constructs (Drummond and Brough, 2016). The PCS measures goal attainment, cognition, and behavior (e.g., “I visualize my dreams and try to achieve them”); the RCS describes contemplation and simulation of possible future events (e.g., “I imagine myself solving a difficult problem before I actually have to face it”); the SPS focuses on creating a goal-oriented plan of manageable action (e.g., “I break down a problem into smaller parts and do one part at a time”); the PrCS measures the level of anticipation and preparedness (e.g., “I think ahead to avoid dangerous situations”); the ISSS measures advice or feedback from one’s immediate social network (e.g., “I ask others what they would do in my situation”); the ESSS captures the desire to share one’s feelings in order to establish an alliance of empathy and comfort (e.g., “When I’m depressed I get out and talk to others”); and the ACS describes passive actions that seek to elude stressful events (e.g., “When I have a problem I like to sleep on it”).

### Assumptions and Data Analytic Plan

Since Study 1 showed that GTS correlated significantly with all facets of HEXACO (see Table 1) and that personality could explain 29% of the variance in PSS (see Table 2), we decided to now model GTS together with SES, IUS, and the multidimensional PCI measure, this time however without the HEXACO measure in the model. The effects of potentially important coping strategies are now tested using PCI (the Proactive Coping Inventory, consisting of the seven subscales; Greenglass et al., 1999). It is reasonable to assume that GTS will be positively related to PCI due to the similarities and possible relationship with social support. Furthermore, PCI has been reported to promote a positive mood (Schwarzer and Taubert, 2002) and alleviate psychopathological outcomes (Greenglass and Fiksenbaum, 2009). On an aggregated level, we believe that PCI will be negatively related to PSS. It remains to be seen which of the specific PCI subscales may predict PSS.

Since we firstly see GTS as rather similar to the A trait and, secondly, believe that specific proactive coping strategies and behaviors may be learned and depend on situations, we treat PCI as a lower-ordered construct compared to GTS. Consequently, PCI is added in the fourth and last hierarchical steps. We conjecture that the relationship between GTS and PSS may be weaker when modeled hierarchically foremost together with the higher-order construct of IUS, but also together with the lower-ordered construct of PCI. We furthermore expect that controlling for SES (Age, Gender, Education, and Income) will influence the effects of IUS, GTS, and PCI on PSS, based on the assumption that education and income also have the potential to alleviate perceived stress (Redmond et al., 2013; Algren et al., 2018).

All the measured constructs are entered in a hierarchical regression model. SES is entered first, IUS in the second, and GTS in the third step. Since specific proactive coping strategies and behaviors may be learned and GTS is similar to the A factor, PCI is here assumed to be a lower-order construct compared to GTS. Consequently, PCI is added in the fourth and last step of the model.

### Results

As may be seen in Table 3, the bi-variate relations from Study 1 are replicated, as a positive significant correlation is found between PSS and IUS and a negative significant correlation between GTS and PSS. The subscales of PCI display rather strong significant negative correlations to PSS, with the exceptions of the uncorrelated SSS and the weakly positively correlated ACS. Furthermore, IUS shows relatively small correlations with the subscales of PCI. It should be noted that there are positive significant correlations between GTS and each subscale of PCI, suggesting that the more people engage in proactive coping the higher the levels of trust is observed and vice versa.

**Table 3:** Means (*M*), standard deviation (*SD*), skewness (Sk), kurtosis (Ku) and Pearson correlations of study variables.

Variables	*M*	*SD*	Sk	Ku	1	2	3	4	5	6	7	8	9	10
1. PSS-10	1.76	0.74	−0.03	2.69	**0.88**									
2. IUS-12	2.65	0.82	0.43	2.76	0.53^***^	**0.90**								
3. PCS	2.97	0.48	−0.47	3.44	−0.33^***^	−0.13^***^	**0.86**							
4. RCS	3.02	0.55	−0.65	3.94	−0.15^***^	0.12^***^	0.65^***^	**0.89**						
5. SPS	2.99	0.62	−0.59	3.43	−0.19^***^	0.10^**^	0.59^***^	0.69^***^	**0.76**					
6. PrCS	2.94	0.59	−0.60	3.37	−0.26^***^	0.06	0.62^***^	0.71^***^	0.67^***^	**0.88**				
7. ISSS	2.83	0.65	−0.42	2.97	−0.01	0.13^***^	0.44^***^	0.52^***^	0.49^***^	0.52^***^	**0.90**			
8. ESSS	2.78	0.76	−0.37	2.51	−0.20^***^	−0.02	0.44^***^	0.44^***^	0.45^***^	0.47^***^	0.71^***^	**0.84**		
9. ACS	2.63	0.70	−0.26	2.88	0.07^*^	0.16^***^	−0.02	0.21^***^	0.14^***^	0.14^***^	0.20^***^	0.14^***^	**0.66**	
10. GTS	3.23	0.80	−0.43	3.10	−0.28^***^	−0.13	0.21^***^	0.22^***^	0.24^***^	0.26^***^	0.24^***^	0.28^***^	0.13^***^	**0.88**

Internal consistency reliabilities (Cronbach’s *α*) in bold in the main diagonal. PSS-10, Perceived Stress Scale (short 10-tem version); IUS-12, Intolerance of Uncertainty Scale (short 12-item version); PCS, Proactive Coping Scale (Proactive Coping Inventory); RCS, Reflective Coping Scale (Proactive Coping Inventory); SPS, Strategic Planning Scale (Proactive Coping Inventory); PrCS, Preventive Coping Scale (Proactive Coping Inventory); ISSS, Instrumental Support Seeking Scale (Proactive Coping Inventory); ESSS, Emotional Support Seeking Scale (Proactive Coping Inventory); ACS, Avoidance Coping Scale (Proactive Coping Inventory); GTS, General Trust Scale (revised 5-item version).

* *p* < 0.05;

** *p* < 0.01;

*** *p* < 0.001.

As in Study 1, a four-step hierarchical regression was conducted. As may be seen in Table 4, the effects of the SES variables in step 1 are similar to the results in Study 1, as age, gender, education, and income account for 18% of the variance in PSS. Again, higher age, income, education, and reporting a male gender are associated with lower PSS. In step 2, IUS shows a strong significant association to PSS and the explained variance increases to 36% jointly with SES. This effect of IUS remains in the third step, whereas GTS shows a significant inverse relationship with PSS similar to Study 1. In the fourth and final step, IUS remains a strong predictor of PSS and the seven subscales of PCI add an additional 9% of explained variance. Jointly, SES, IUS, GTS, and PCI now explain 47% of the variance in PSS. The PCI subscales show some overlap in the regression but the Variance Inflation Factors (VIF < 3) do not indicate severe multicollinearity (Hair et al., 2014). The subscales PCS, PrCS, and ESSS show significant inverse effects on PSS, whereas ISSS interestingly indicate a positive effect, when all of the other variables are accounted for, in contrast to the non-significant pairwise correlation between ISSS and PSS. As expected, the effect of GTS is attenuated when modeled together with PCI. While the IUS construct explains the most variance, the lower-ordered PCI adds significantly to the explained variance in PSS. As in Study 1, the explanatory power of GTS is low, but significant and in the expected inverse direction.

**Table 4:** Hierarchical multiple regression model testing effects on the Perceived Stress Scale (PSS-10).

Variables	Model^a^							
	1		2		3		4	
	*β*	*SE*	*β*	*SE*	*β*	*SE*	*β*	*SE*
IUS-12			0.45^***^	0.02	0.44^***^	0.02	0.41^***^	0.02
GTS					−0.14^***^	0.02	−0.08^**^	0.02
PCS							−0.14^***^	0.05
RCS							0.03	0.05
SPS							−0.05	0.04
PrCS							−0.17^***^	0.04
ISSS							0.22^***^	0.04
ESSS							−0.18^***^	0.03
ACS							0.02	0.03
Age	−0.34^***^	0.01	−0.21^***^	0.01	−0.18^***^	0.01	−0.19^***^	0.01
Gender^b^	0.15^***^	0.04	0.12^***^	0.04	0.11^***^	0.04	0.12^***^	0.04
Education	−0.05	0.02	−0.06^*^	0.01	−0.03	0.01	0.01	0.01
Income	−0.09^**^	0.01	−0.08^**^	0.01	−0.07^*^	0.01	−0.05	0.01
*_Adj_R*^2^	0.18^***^		0.36^***^		0.38^***^		0.47^***^	
*Δ_Adj_R*^2^			0.18^***^		0.02^***^		0.09^***^	
*F*	60.83^***^		124.88^***^		112.29^***^		74.76^***^	

PSS-10, Perceived Stress Scale (short 10-tem version); IUS-12, Intolerance of Uncertainty Scale (short 12-item version); PCS, Proactive Coping Scale (Proactive Coping Inventory); RCS, Reflective Coping Scale (Proactive Coping Inventory); SPS, Strategic Planning Scale (Proactive Coping Inventory); PrCS, Preventive Coping Scale (Proactive Coping Inventory); ISSS, Instrumental Support Seeking Scale (Proactive Coping Inventory); ESSS, Emotional Support Seeking Scale (Proactive Coping Inventory); ACS, Avoidance Coping Scale (Proactive Coping Inventory); GTS, General Trust Scale (revised 5-item version).

a Perceived Stress Scale (PSS-10) modeled as dependent variable.

b Female = 1.

* *p* < 0.05;

** *p* < 0.01;

*** *p* < 0.001.

### Discussion

We conclude that, in contrast to Study 1, IUS now shows a stronger relationship to PSS and explains a larger part of the variance in PSS and that this association largely is in line with previously reported studies (Crum et al., 2013). Since the GTS construct again adds significantly to the explained variance of PSS even after controlling for strong factors such as intolerance of uncertainty and socio-economic background, we conclude that trust shows a unique, albeit small, effect under control for interrelated constructs. However, the results indicate that the predictive power of trust on perceived stress is attenuated when proactive coping is introduced. This is not surprising since the PCI subscales contain two accounts of social support, different behaviors, and coping strategies, that involve respective others and social connectedness to a large degree. The PCI seems to add a unique effect contributing to perceived stress above and beyond the effects of SES, IUS, and GTS. An inverse association between PSS and a coping inventory such as PCI should be expected (Schwarzer and Taubert, 2002; Greenglass and Fiksenbaum, 2009). Additionally, the PCI has been reported to be positively related to the personality trait of E and C and negatively related to Neuroticism (Straud et al., 2015). In a similar fashion, C and O have been reported to be positively related to trustworthiness while Neuroticism has been found to correlate negatively with trust (Evans and Revelle, 2008). It is thus suggested that proactive coping and trust may be comparable in terms of their associations and possibly predictive validity in explaining stress-related outcomes.

Although we acknowledge that the hierarchical order in which the constructs of GTS and PCI theoretically could be structured is up for debate, we interpret our results as suggesting that trust or social support can be understood as a subset of proactive coping. Yet, the many different subscales of the PCI should be expected to account for more variance since it consists of more coping constructs than social support. All subscales of PCI in fact show significant inverse associations to PSS with the interesting exception of ISSS which relates positively to stress. This may reflect that the subscales of PCI are conditional on each other or that one of the subscales acts as a suppressor variable in the regression (e.g., MacKinnon et al., 2000). Overall, the results from Study 2 largely confirm the patterns from Study 1 in that trust, with the addition of proactive coping, can reduce stress over and above what can be explained by personality and socio-economic variables.

## General Discussion

In two studies, we model how the constructs of general trust (GTS), proactive coping (PCI, in Study 2), personality (HEXACO, in Study 1), and intolerance of uncertainty (IUS), jointly relate to perceived stress (PSS) while controlling for SES. Our aim is to investigate whether a measure of general trust (GTS) and a measure of proactive coping (PCI) can explain variance in perceived stress (PSS) over and above what background variables (SES), personality (HEXACO), and IU (IUS) can explain. We model these constructs hierarchically based on the assumption that there are higher-order constructs that are less context-dependent and more related to neurobiological underpinnings and lower-order constructs that are more context-dependent in relation to specific situations and behaviors (DeYoung, 2010, 2013).

In line with the stress-buffering hypothesis of social support (Cohen and Wills, 1985), we suggest that general trust may function as a coping mechanism in alleviating psychological stress. In both our studies, we find the expected negative relationship between general trust and perceived stress. A number of studies substantiate this finding in showing that trust and stress are inversely related (Takahashi et al., 2005; Cardoso et al., 2013; FeldmanHall et al., 2015; McQuaid et al., 2016) and that stress may lead people to seek out more prosocial interactions (von Dawans et al., 2012; Raposa et al., 2015). Other studies have furthermore shown positive relations between trust and active appraisal and coping strategies (Buchwald, 2003; Wilson and Darke, 2012).

Given this, there is a case to be made for the reactivity of trust as a coping mechanism in that when faced with stressful situations, individuals as a response seem to upregulate their level of trust (Yanagisawa et al., 2011; Koranyi and Rothermund, 2012). The positive association between general trust and proactive coping found in the present research is therefore not surprising, especially since the proactive coping inventory in part consists of social support. Yet, it was not clear from the onset of this research how the subscales of proactive coping would be related to general trust. Our findings show that general trust was significantly correlated with each subscale and that the ACS showed the weakest relationship, which is in line with the idea that higher trust is related to more engaged coping strategies (Buchwald, 2003; Wilson and Darke, 2012).

In light of our specified confounding expectation, we find support for the notion that the effect of general trust on perceived stress is attenuated when modeled hierarchically with other interrelated constructs such as the personality inventory of HEXACO, the dispositional construct of IU, the proactive coping inventory of PCI and SES. It is well-known that personality relates to stress sensitivity along with other psychopathologies (Carver and Connor-Smith, 2010; Kotov et al., 2010; Ebstrup et al., 2011), and we find in line with such previous research that, in particular, emotional stability is positively and X is negatively related with perceived stress.

The relation between IU and stress is however researched to a lesser degree, as most studies on IU focus on anxiety and mood disorders (Carleton, 2016). While a study by Crum et al. (2013) reports a positive association between IU and perceived stress, our research is to our knowledge novel in modeling this specific relationship together with other interrelated constructs. We find this a bit surprising, firstly since it may be intuitive that uncertainty can lead to both subjective and physiological stress responses (De Berker et al., 2016), and secondly, since IU may be linked to different behavioral outcomes (see Birrell et al., 2011 for a review). In contrast to personality traits, the IU measure (IUS) could thus function as a short and specific instrument for measuring dispositional susceptibility to anxiety and stress.

When interpreting our general findings, we conclude that there are several potential buffers to perceived psychological stress, including SES (Redmond et al., 2013; Algren et al., 2018). Specifically, in Study 1 and Study 2 we show that lower perceived stress is associated with background variables such as reporting a male gender, higher age, education, and income. However, when modeled jointly with the constructs of personality, IU, general trust, and proactive coping, the effects of these robust background variables are found to be attenuated. Given this, we consider the presented regression models as a way of showing non-confounded effects.

The awareness of the positive health effects of trust is increasing in public health and social medicine (Kawachi et al., 2008; Giordano and Lindström, 2016). Recent research has requested that future studies should include similar and interrelated constructs in order to control, explain, and disentangle alleged bi-variate relationships between trust and health (Shiell et al., 2020). In response to these requests, we find the alleviating effects of trust on perceived stress to be small but robust. However, given the relatively weak correlations and small amounts of explained variance found it may be questioned whether trust can be effectively increased through programs and policy measures. In our data, the effects of general trust are to the largest part explained by beneficial combinations of constructs that may be more dispositional. Furthermore, although we find that trust is positively related to all the measured subscales of coping and that the effect of trust is attenuated when coping is introduced in our regression model, it may still be questioned if, and to what degree, general trust really can function as a coping mechanism. Assuming that trust at least partly is a context-sensitive construct, we still conjecture that uncertain situations may likely elicit increased levels of trust in relevant experts, professions, and institutions, thus serving as a coping mechanism. Successfully promoting people’s health by reducing psychological stress through increased trust therefore requires knowledge of the interactions between general and situational trust (Han, 2019). Whether such reactions to uncertainty could or should be seen as coping is however still up for debate.

### Limitations

The present results are based on data consisting of two samples taken from a pool of web panels of American citizens provided by Qualtrics. Web panels comprised of self-recruited participants who may suffer from selection bias. However, such biases in population sampling are hard to overcome unless mandatory Census programs are used. Another limitation is that we only use self-report instruments in a cross-sectional design. Therefore, we cannot ascertain how the measured constructs relate, for example, to observer ratings, physiological indicators, behavioral data, or causal patterns across the lifespan.

### Future Directions

Our results indicate that dispositional constructs such as personality and IU contribute largely, and that general trust and proactive coping contribute to a lesser degree, in explaining stress. In order to explore the possible stress benefits of trust, experimental studies conducting pre‐ and post-measures of stress should investigate the efficacy of stress-prevention measures based on attempts to increase general trust and proactive coping. Another endeavor is to investigate the degree to which general trust and proactive coping strategies can alleviate stress for people with different combinations of personality traits. It has furthermore been suggested that much of the earlier research studying the association between general trust and health outcomes are too simplistic and treat general trust as a natural phenomenon. Instead, the link between general trust and health should be investigated from a wider systems perspective acknowledging that cultural differences may be important in explaining this relationship. The causal mechanisms between general trust, coping, stress, and health are far from clear and need to be further investigated.

### Conclusions

Overall, stress is one of the most critical antecedents of negative health outcomes. Assumed to correlate with lower morbidity and mortality, general trust has increasingly been seen as a quick-fix or buffer to psychological stress. We have shown that the negative relationship between trust and stress is robust but weak and significantly reduced when modeled jointly with personality, proactive coping, and socioeconomic status. Our findings suggest that general trust may, to a large degree, be considered a proxy for a beneficial combination of individual characteristics. When studying general trust as a remedy for stress, it is, therefore, reasonable to question what underlies the often overly optimistic health outcomes associated with general trust.

## Data Availability Statement

The raw data supporting the conclusions of this article will be made available by the authors, without undue reservation.

## Ethics Statement

Ethical review and approval was not required for the study on human participants in accordance with the local legislation and institutional requirements. The patients/participants provided their written informed consent to participate in this study.

## Author Contributions

AC has the main responsibility for this manuscript. He, together with L-OJ, conceptualized the idea and planning of the study within the research project for which this manuscript is part of the project. AC conducted the data collection, analysis, and a large part of the writing of this manuscript. L-OJ has contributed largely to theoretical development, the structure of the manuscript, reviewing, and writing. Both the authors contributed to the article and approved the submitted version.

### Conflict of Interest

The authors declare that the research was conducted in the absence of any commercial or financial relationships that could be construed as a potential conflict of interest.
